# Efficacy and safety of human umbilical cord-derived mesenchymal stem cells (hUC-MSC PLEB001) for the treatment of grade II–IV steroid-refractory acute graft-versus-host disease: a study protocol for a multicenter, randomized, double-blind, placebo-controlled, phase II trial

**DOI:** 10.1186/s13063-023-07305-0

**Published:** 2023-05-03

**Authors:** Donglin Yang, Xiaoqiang Hou, Kun Qian, Yuhang Li, Liangding Hu, Liang Li, Mingzhe Han, Chen Yao, Daihong Liu

**Affiliations:** 1 State Key Laboratory of Experimental Hematology, National Clinical Research Center for Blood Diseases, Haihe Laboratory of Cell Ecosystem, Institute of Hematology & Blood Diseases Hospital, Chinese Academy of Medical Sciences & Peking Union Medical College, Tianjin, China; 2 Platinumlife Biotechnology (Beijing) Co., Ltd., Beijing, China; 3 School of Medicine, Nankai University, Tianjin, China; 4 Department of Hematology, the Fifth Medical Center of Chinese PLA General Hospital, Beijing, China; 5 Peking University Clinical Research Institute, Peking University First Hospital, Beijing, China

**Keywords:** Acute graft-versus-host disease, Mesenchymal stem cell, Steroid, Second-line therapy, Human umbilical cord, Protocol

## Abstract

**Background:**

Systemic corticosteroid therapy failure is quite common in patients with newly diagnosed acute graft-versus-host disease (aGVHD). Growing evidence has suggested that mesenchymal stem cell (MSC) therapy could be a promising treatment option for aGVHD due to its distinctive immunomodulating functions. However, there is a lack of randomized well-controlled clinical trials.

**Methods:**

This is a clinical trial protocol for a multicenter, randomized, double-blind, placebo-controlled phase II study. The aim of the trial is to evaluate the efficacy and safety of the administration of the human umbilical cord-derived MSC product hUC-MSC PLEB001 in patients with grade II–IV, steroid-refractory aGVHD. A total of 96 patients will be randomized 1:1 to receive MSC or placebo treatment twice per week for 4 weeks, in addition to second-line therapy according to institutional standards. Patients who achieve partial response (PR) at day 28 will be eligible to receive further infusions twice per week for an additional 4 weeks.

**Discussion:**

This study will evaluate the efficacy and safety of MSC therapy in patients who have failed first-line steroid treatment for grade II–IV aGVHD.

**Trial registration:**

Chinese Clinical Trial Registry (ChiCTR), ChiCTR2000035740. Registered on 16 August 2020.

**Supplementary Information:**

The online version contains supplementary material available at 10.1186/s13063-023-07305-0.

## Background

Allogeneic hematopoietic stem cell transplantation (allo-HSCT) is a potential cure for various hematologic diseases [1]. Acute graft-versus-host disease that involves the skin, liver, or gastrointestinal tract is one of the common complications following allo-HSCT, with an overall incidence of 30–50% [2]. Systemic glucocorticoids, i.e., 1–2 mg/kg methylprednisolone, have been considered the first-line therapy for newly diagnosed aGVHD [3, 4]. However, steroid refractoriness, including steroid resistance and steroid dependence, occurs in more than 40% of patients and is associated with dismal outcomes [5, 6].

The use of most second-line therapies for aGVHD has been off-label, including basiliximab, α1-antitrypsin, and extracorporeal photopheresis [7]. Overall survival of aGVHD is not improved by most second-line therapies [8] due to immunosuppression-associated infections. Ruxolitinib, a Janus kinase (JAK) inhibitor, was approved for the treatment of steroid-refractory aGVHD by the US Food and Drug Administration (FDA) in May 2019 [9, 10]. However, this drug failed to demonstrate a significant advantage in terms of overall survival (OS) and raised concerns of hematologic toxicity and virus reactivation [11].

Mesenchymal stem cell therapy, which was developed in 2004, offers a new option for the treatment of aGVHD with few side effects. In the allo-HSCT setting, MSCs have been found to be effective for GVHD via immunomodulation, such as inhibiting the proliferation of T cells, promoting the expansion of Treg cells, and regulating the production of soluble factors such as NO and IDO [12, 13]. In addition, HLA-DR is not expressed on MSCs, which show minimal risk of initiating an allogeneic immune response [14]. In 2004, for the first time, Le Blanc et al. treated a patient with grade IV refractory aGVHD involving the gut and liver by infusion of MSCs from a haploidentical donor [15]. In 2008, Le Blanc et al. reported a phase II multicenter prospective clinical trial. Fifty-five patients with grade II–IV steroid-refractory aGVHD received MSCs from HLA-identical sibling donors, haploidentical donors, or third-party HLA-mismatched donors. The results showed that 54.5% of patients achieved complete response, 16.4% showed improvement, and no patients experienced side effects during or after infusion. The complete responders demonstrated superior 2-year overall survival after transplantation (53% vs. 16%, *p* = 0.018) [16]. Since then, the number of studies applying MSCs to treat steroid-refractory aGVHD has increased rapidly. However, there were apparent inconsistencies in outcomes between these trials, which were related to variability in MSC sources, production methodology, and limitations in study design [17].

Hence, there is an urgent need for randomized controlled trials (RCTs) to confirm the effect of MSCs on steroid-refractory aGVHD. By December 2021, nine RCT studies had been registered on ClinicalTrials.gov or the WHO’s international clinical trial registration platform (ICTRP) [18]. In 2020, Kebriaei et al. reported a multicenter, randomized, phase 3, double-blind, placebo-controlled trial of MSCs for 260 steroid-refractory patients [19]. The study failed to meet its primary endpoint—there was no significant difference between the durable complete response (DCR) rate in patients treated with MSCs in comparison to those treated with placebo (35% vs. 30%, *p* = 0.42). The trial found that MSC treatment led to significantly improved overall response (OR, 55% vs. 26%, *p* = 0.05) and DCR rates (29% vs. 5%, *p* = 0.047) in patients with liver aGVHD. There was also a higher overall response rate in children treated with MSCs than in controls (64% vs. 23%, *p* = 0.05). However, this study design involved both pediatric and adult patients, leading to clinical heterogeneity. Another limitation of the study was the nonstandardized use of second-line agents across study units.

Therefore, we designed a prospective, randomized, double-blind, placebo-controlled, multicenter phase II clinical trial to evaluate the efficacy and safety of the human umbilical cord-derived MSC product hUC-MSC PLEB001 in patients with grade II–IV steroid-refractory aGVHD.

## Methods

### Trial design

This is a multicenter, randomized, double-blind, placebo-controlled, superiority, phase II trial (Chinese Clinical Trial Registry, ChiCTR2000035740) that will enroll patients with grade II–IV steroid-refractory aGVHD from 8 Chinese centers. The primary endpoint is the overall response rate (ORR) at day 28. A total of 96 patients will be randomly assigned at a 1:1 ratio to receive either MSCs or placebo in a double-blinded manner. To ensure the interests of the patients, both the MSC and placebo groups will receive conventional second-line therapy. The MSC group will receive an infusion of hUC-MSC PLEB001 at 1.0 × 10^6^ MSC/(kg of subject, adjusted body weight) twice a week for 4 weeks. The control group will receive placebo treatment at the same frequency. The treatment efficacy will be assessed on the 28th day after the first infusion in all patients. The treatment will be terminated if the patient reaches complete response (CR), no response (NR), or progression of disease (PD) on the 28th day. Patients who display PR at day 28 will receive further infusions twice per week for an additional 4 weeks. The total treatment period will last no more than 8 weeks.

The conventional second-line therapy for patients with steroid-refractory aGVHD includes basiliximab, ruxolitinib, mycophenolate mofetil, methotrexate, etc. Investigators from each center will ensure the consistency of indications, category, and administration of second-line therapies. If deterioration or rapid progression of aGVHD is observed, another second-line agent can be added to the trial treatment regimen.

The protocol and the proposed informed consent form (ICF) will be reviewed and approved by an independent ethics committee (EC) before the study starts. Eligible patients may only be enrolled in the study after providing written informed consent.

In addition, peripheral blood samples will be collected from patients for the detection of Treg and Th17 cell populations prior to and after the treatment. Additional consent for peripheral blood sample collection and detection will also be included in the informed consent form.

### Study procedures and assessment

Investigators will introduce the trial to patients, who will be shown video and advertisement sheets describing the main aspects of the trial. Before obtaining informed consent, investigators must provide sufficient explanation to ensure that patients understand the nature, scope, and consequences of the clinical trial.

**Fig. 1:**
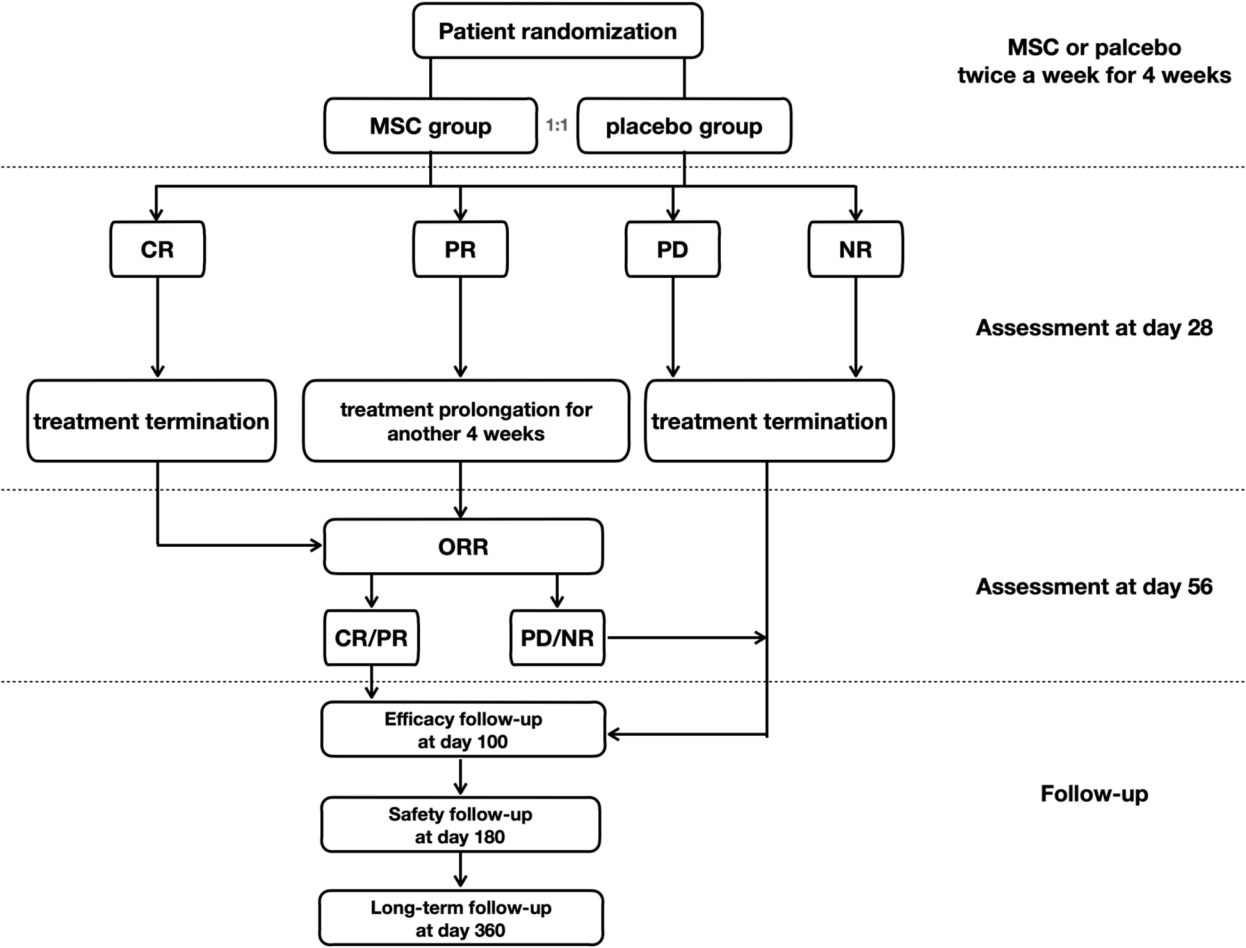
Flowchart of the study

This trial will include a 7-day screening period, a treatment period lasting 4 to 8 weeks, and a follow-up period. The follow-up will continue until death, withdrawal from the study, or 360 ± 15 days after the first infusion. The flowchart of the study is presented in Fig. 1, and the schedule of screening, intervention. and follow-up are shown in Table 1.

**Table 1:** Schedule of screening, intervention, and follow-up

Stage		Screening	Treatment																		Follow-up			
**Visit**		**Visit 1**	**Visit 2**	**Visit 3**	**Visit 4**	**Visit 5**	**Visit 6**	**Visit 7**	**Visit 8**	**Visit 9**	**Visit 10**	**Visit 11**	**Visit 12**	**Visit 13**	**Visit 14**	**Visit 15**	**Visit 16**	**Visit 17**	**Visit 18**	**Visit 19**	**Visits 20–24**	**Visit 25**	**Visit 26**	**Visit 27**
**Time (day)**		** − 14 ~ 0**	**1**	**4 ± 1**	**8 ± 1**	**11 ± 1**	**15 ± 1**	**18 ± 1**	**22 ± 1**	**25 ± 1**	**28 ± 1**	**29 ± 1**	**32 ± 1**	**36 ± 1**	**39 ± 1**	**43 ± 1**	**46 ± 1**	**50 ± 1**	**53 ± 1**	**56 ± 1**	**63 ± 3, 70 ± 3, 77 ± 3, 84 ± 3, 91 ± 3**	**100 ± 3**	**180 ± 7**	**360 ± 15**
**Time (week)**		**0**	**W1**	**W1**	**W2**	**W2**	**W3**	**W3**	**W4**	**W4**	**W4**	**W5**	**W5**	**W6**	**W6**	**W7**	**W7**	**W8**	**W8**	**W8**	**W9, W10, W11, W12, W13**	**W14**	**W26**	**W52**
Informed consent		**X**																						
Demographic data		**X**																						
Medical history		**X**																						
Vital signs		**X**	**X**	**X**	**X**	**X**	**X**	**X**	**X**	**X**	**X**	**X**	**X**	**X**										
Randomization		**X**													**X**	**X**	**X**	**X**	**X**	**X**	**X**	**X**	**X**	
Colonoscopy		**X**																						
Physical exam		**X**									**X**									**X**				
Height/weight		**X**	**X**	**X**	**X**	**X**	**X**	**X**	**X**	**X**	**X**	**X**	**X**	**X**	**X**	**X**	**X**	**X**	**X**	**X**				
Laboratory test^a^	CBC	**X**	**X**	**X**	**X**	**X**	**X**	**X**	**X**	**X**	**X**	**X**	**X**	**X**	**X**	**X**	**X**	**X**	**X**	**X**				
	Comprehensive metabolic panel	**X**	**X**	**X**	**X**		**X**		**X**		**X**		**X**		**X**		**X**			**X**	**X**	**X**		
	CRP	**X**																						
	Coagulation	**X**		**X**	**X**		**X**		**X**		**X**									**X**				
	Blood type	**X**																						
	HBV, HCV, TP, HIV serostatus	**X**			**X**		**X**		**X**		**X**		**X**		**X**		**X**			**X**				
	CMV-DNA	**X**			**X**		**X**		**X**		**X**		**X**		**X**		**X**			**X**				
	EBV-DNA	**X**			**X**		**X**		**X**		**X**		**X**		**X**		**X**			**X**				
	Urinalysis	**X**	**X**	**X**	**X**		**X**		**X**		**X**		**X**		**X**		**X**			**X**				
	Fecal analysis and OBT	**X**	**X**	**X**	**X**		**X**		**X**		**X**		**X**		**X**		**X**			**X**				
12-leads ECG^a^		**X**									**X**									**X**				
Abdominal ultrasound scan^a^		**X**									**X**									**X**				
Th17 and Treg count (prior to MSC infusion)			**X**				**X**				**X**									**X**				
CEA, CA125, CA199, AFP^a^		**X**									**X**									**X**				
X-ray or CT of chest		**X**									**X**									**X**				
Gastrointestinal examination		**X**		**X**	**X**		**X**		**X**		**X**		**X**		**X**		**X**			**X**	**X**	**X**		
Cutaneous examination		**X**		**X**	**X**		**X**		**X**		**X**		**X**		**X**		**X**			**X**	**X**	**X**		
aGVHD evaluation^1^		**X**		**X**	**X**		**X**		**X**		**X**		**X**		**X**		**X**			**X**	**X**	**X**		
ECOG score		**X**									**X**									**X**		**X**		
Pregnancy test( for female of child bearing potential)		**X**									**X**									**X**				
SaO2/SAT^2^		**X**	**X**	**X**	**X**	**X**	**X**	**X**	**X**	**X**		**X**	**X**	**X**	**X**	**X**	**X**	**X**	**X**					
Inclusion/exclusion criteria		**X**																						
Intervention			**X**	**X**	**X**	**X**	**X**	**X**	**X**	**X**		**X**	**X**	**X**	**X**	**X**	**X**	**X**	**X**					
AEs			**X**	**X**	**X**	**X**	**X**	**X**	**X**	**X**	**X**	**X**	**X**	**X**	**X**	**X**	**X**	**X**	**X**	**X**	**X**	**X**	**X**	
Concomitant disease and medication		**X**	**X**	**X**	**X**	**X**	**X**	**X**	**X**	**X**	**X**	**X**	**X**	**X**	**X**	**X**	**X**	**X**	**X**	**X**	**X**	**X**	**X**	
Medical recording of aGVHD after investigational product																					**X**	**X**	**X**	
Imaging of secondary tumors																						**X**	**X**	**X**
Survival status			**X**	**X**	**X**	**X**	**X**	**X**	**X**	**X**	**X**	**X**	**X**	**X**	**X**	**X**	**X**	**X**	**X**	**X**	**X**	**X**	**X**	**X**

Subjects who withdraw from the trial should be assessed according to visit 19

Detailed information about the test items is shown in the clinical trial protocol V6.0, which could be provided by the trial sponsor

1. According to the modified Glucksberg criteria

2. Monitoring with a pulse oximeter at least 30 min before MSC infusion to 2 h after infusion

^a^Test results prior to randomization are acceptable during the screening period, and the exact time limits are described in the clinical trial protocol V6.0

Subjects who deviate from intervention protocols or the follow-up schedule of assessments will follow the same calendar as those with good compliance, except adherence assessment.

Participants may withdraw from the study for any reason at any time. Investigators will make the best effort to evaluate patients based on visit 19 before they withdraw from the trial. Detailed reasons for withdrawal, concomitant medications, and adverse events will be recorded to obtain safety data for those who withdraw consent or are lost to follow-up.

### Study setting

The study will be conducted in 8 hematopoietic stem cell transplantation centers in China, including the First Medical Center of Chinese PLA General Hospital; the Fifth Medical Center of Chinese PLA General Hospital; Institute of Hematology & Blood Disease Hospital, Chines Academy of Medical Sciences & Peking Union Medical College; Shandong Provincial Hospital Affiliated to Shandong First Medical University; the Second Hospital of Hebei Medical University; the First Affiliated Hospital of Zhengzhou University; Henan Provincial People’s Hospital; and the Affiliated Hospital of Xuzhou Medical University, which are highly experienced in the management of GVHD and hematopoietic stem cell transplantation with routine patient education about clinical trials.

This study started in June 2020, and patients will be recruited over the course of 2 years. Each study site will use the newly diagnosed aGVHD patient list to identify and recruit potential subjects. Recruitment videos and advertisements for potential subjects will be advertised among patients through their doctors. If a patient intends to enter the study, dedicated staff will meet with the patient face-to-face, give more detail to introduce the study, and arrange the appropriate screening procedures.

### Study endpoints

This trial aims to assess the efficacy and safety of hUC-MSC PLEB001 in patients with grade II–IV steroid-refractory aGVHD. The primary endpoint is the overall response rate (ORR) at day 28, which is defined as the proportion of patients achieving a CR or PR without the use of additional systemic therapies for aGVHD. The secondary endpoints include the CR rate at day 28, ORR at day 56, CR rate at day 56, durable ORR at day 56, DCR, OS, and Eastern Cooperative Oncology Group (ECOG) score. The safety endpoints include adverse events (AEs), serious adverse events (SAEs), and infusion toxicity (Table 2).

**Table 2:** Study endpoints

Aim	Indicator
Primary endpoint	ORR at day 28
Secondary endpoints	CR at day 28, day 56
	ORR at day 56
	Durable ORR at day 56
	DCR within day 100
	OS at day 100, day 180
	ECOG score at day 28, day 56, day 100
Safety endpoints	AE/SAE at day 180
	infusion toxicity at day 14

1. Complete remission (CR): absence of any aGVHD-related symptoms

2. Partial remission (PR): an improvement in severity of at least 1 stage in all initial organs without further deterioration of other organs

3. No response (NR): the absence of improvement or deterioration of symptoms in any organ or death

4. Progressive disease (PD): a deterioration in the severity of at least 1 stage in a single organ with or without improvement in other organs

5. Overall response rate (ORR) = CR + PR

6. Durable ORR: the percentage of subjects who achieve CR + PR on day 28 and remain CR + PR on day 56

7. Durable complete response (DCR): complete resolution of aGVHD symptoms for at least 28 days within 100 days after the first infusion

8. Infusion toxicity includes fever, phlebitis, headache, dizziness, nausea, chest pain, shortness of breath, transient pulmonary edema, allergic reaction, skin rash, injection site pruritus, etc.

### Study population

The target population will be patients suffering grade II–IV steroid-refractory aGVHD.

The inclusion criteria are as follows:

i. Male or female aged between 13 and 70 years
ii. Patients with malignant hematologic diseases who received their first allogeneic hematopoietic stem cell transplantation and developed grade II–IV steroid-refractory aGVHD. AGVHD may develop after donor lymphocyte infusion

First-line glucocorticoid therapy refers to methylprednisolone 1 mg/kg/day or 2 mg/kg/day or an equivalent dose of steroids.

Steroid-refractory aGVHD is defined as patients administered first-line steroid therapy who exhibit one of the following responses: disease progression after 3 days, failure to achieve a PR after 7 days, failure to achieve CR after 14 days of steroid treatment (categorized as steroid resistance), or reactivation of aGVHD during tapering of steroids (categorized as steroid dependence).

iii. Patients are able to be treated with the trial drug within 3 days after enrollment
iv. Prior to the trial, the subject or his or her legal representative (for subjects < 18 years of age) is capable of providing written informed consent

The exclusion criteria are as follows:

i. Patients with lung disease judged by the investigator to be unfit to participate in the trial.
ii. Serologically positive for HCV-Ab, TP-Ab, and HIV-Ab
iii. Patients with sinusoidal obstruction syndrome
iv. Patients showed mental status changes due to brain lesions or other medical causes since the onset of aGVHD
v. Patients with cytomegalovirus (CMV) enterocolitis, diarrhea due to transplant-associated thrombotic microangiopathy, or gastrointestinal infection
vi. Patient creatinine clearance < 30 mL/min, calculated by the Cockcroft-Gault formulation
vii. ECOG score > 3
viii. Patients with evidence of life-threatening complications within 6 months prior to enrollment that would interfere with the outcome evaluation of the study, including but not limited to uncontrolled infection, pulmonary hypertension, severe cardiac failure (NYHA class III and IV), unstable angina or acute myocardial infarction, and refractory hypertension
ix. Patients with active malignant solid tumors within 5 years prior to the study, except for radically treated cervical cancer, in situ limited prostate cancer and nonmelanoma skin cancer
x. Patients with mental or neurological disorders
xi. Patients who received systemic first-line treatment other than glucocorticoids for aGVHD prior to screening
xii. Patients with a history of severe allergy to blood products or a history of allergy to allogeneic proteins
xiii. Female patients who are lactating or planning a pregnancy or egg donation during the study and the follow-up period or male patients who have plan to father a child or donate sperm during the study and the follow-up period
xiv. Participation in other clinical trials within the previous 1 month

### Investigational product

hUC-MSC PLEB001 comprises human umbilical cord-derived mesenchymal stem cells that have been ex vivo cultured and formulated in saline supplemented with human serum albumin. Saline will be used as the placebo and provided in the same packaging form as hUC-MSC PLEB001.

The investigational product will be administered by peripheral intravenous drip as follows: according to the transfusion routine, a peripheral intravenous drip, using a disposable blood transfusion set, will be conducted for investigational product administration. The initial 10 ml will be dripped at approximately 2 ml/min, and the residual volume will be dripped at 4 ml/min. After the infusion, the pipeline will be flushed with saline.

### Randomization and double-blind design

To reduce the possible selection bias and efficacy evaluation bias from investigators and subjects, this trial will adopt a randomized, double-blind, placebo-controlled design. Since the baseline treatment is the current standard treatment regimen, this study will adopt a loading design, which meets the ethical requirements.

The multicenter subblock randomization method will be used, and a random number table will be generated by professional statisticians using SAS (version 9.4) software. The subjects will be randomly allocated, and the investigational drugs will be allocated in the Central Randomization System of Clinical Information Management System (CIMS-CRS).

After signing the informed consent form, each subject will be given a screening number. The length of the screening number is N, and the naming rule is “S” + center number (XX) + sequence number (XXX). The random number is obtained after successful randomization, the length of the random number is N, and the naming rule is N-digit natural number (XXX).

In this trial, CIMS-CRS will be connected with the Alibaba Health precision traceability system to complete drug blinding. The unique tracking numbers for investigational drug distribution are generated by the drug management module of the Alibaba Health precision traceability system. The two systems work together to accurately manage and fully trace investigational drugs and clinical data.

A unique traceability code on the subject’s medication bag must be recorded using a code scanner prior to use of all medications. Normally, blinding should not be broken until all subjects have completed the study and the database is locked. To ensure the double-blind design, a light-protected infusion set will be used to shield the investigational product.

If severe safety issues arise in the study that requires the investigator to know the subject’s group, an unblinding procedure should be initiated. An emergency unblinding license will be generated in the center system, which permits the investigators and subjects to check their grouping. Immediately upon unblinding, the sponsor and the contract research organization need to be notified. Information about unblinding should be recorded in the subject’s source documentation and electronic medical record.

### Safety reporting

#### AE

Adverse events are collected through interrogation, physical examination, laboratory or other tests, and self-reporting. Investigators are required to collect AEs from the time of the first dose and continue to collect AEs until 180 days after the first infusion, death, or early withdrawal from the clinical study (whichever occurs first).

In this trial, the severity of adverse events is graded according to the common terminology criteria adverse events (CTCAE 5.0) published by the National Cancer Institute (NCI) [20]. The potential association between adverse events and investigational product will be examined based on the “Adverse Drug Reaction Reporting and Detection Workbook” issued by the National Adverse Drug Reaction Monitoring Centre.

#### SAE

The collection and assessment of serious adverse events will be consistent with the procedures for adverse events. To ensure patient safety, every SAE, regardless of suspected causality, that occurs after the patient provides informed consent and until the end of treatment visit, will be reported to the sponsor and ethics committee within 24 h of learning of its occurrence.

Some events requiring hospitalization or prolonged hospitalization may not be treated as SAEs, including hospitalization or prolonged hospitalization for reasons other than AEs and hospitalization for a surgery scheduled prior to the trial, but these must be clearly documented in the medical record.

### Concomitant medication

Cyclosporine A, mycophenolate mofetil, and short-term methotrexate for aGVHD prophylaxis will be administered. Subjects may be given antibiotics to prevent or treat infections that occur after transplantation. In addition, subjects will be able to receive supportive therapy. No other trial agents or MSC products will be allowed within 30 days prior to and during the trial.

### Sample size

This study is an exploratory study, so the sample size will not take into account the power associated with statistical hypotheses. Forty randomized subjects in each group will be included in the statistical analysis. Assuming a 20% dropout rate, we plan to enroll 96 subjects (48 in each group).

### Data collection and management

An electronic data management center (EDC) will be used in this trial for data collection, with reliable retention of audit recording and effective management of account privileges. The case report form (CRF) will be designed based on the trial protocol, and the electronic case report form (eCRF) will be created in the EDC. The medical record and original transcript will be preserved and scanned to perform an electronic backup as the source documents for 30 years.

Investigators will be required to collect data on subjects according to Good Clinical Practice (GCP) and study protocol requirements. The investigator will enter the data into the EDC system and preserve it in accordance with the eCRF guidelines.

### Statistical analysis

All analyses will be carried out according to a predetermined statistical analysis plan by utilizing SAS 9.4. Two-tailed tests will be used for most statistical tests, and a *p* value of less than 0.05 will be considered statistically significant. Comparisons between groups for quantitative data will be performed by the independent samples *t* test or Wilcoxon rank sum test. Comparisons between groups of categorical data will be performed by the chi-square test or Fisher’s precision probability test. Comparisons between groups of ranked data will be performed by the Wilcoxon rank sum test or CMH test.

The full analysis set (FAS) is the set of subjects identified according to the intention-to-treat (ITT) principle. It includes all patients who have received at least one dose of study treatment after randomization. The per-protocol set (PPS) consists of the subjects in the FAS who meet the requirements of the trial and are in good compliance. The formula for calculating the adherence of subjects is as follows: adherence rate = (actual infusion dose during follow-up/planned infusion dose) × 100%. Good compliance is defined as ≥ 80% adherence rate. The safety set (SS) includes all patients who received at least one dose of study treatment. The primary and secondary efficacy analyses will be based on both the FAS and PPS. Subjects who present with protocol nonadherence will be analyzed in the FAS and SS.

Adverse events, laboratory tests, symptoms, signs, and the association between adverse events and the trial drug are presented in the form of a table.

The missing values of the primary endpoint will be processed by worst observation carried forward (WOCF). The other missing values will be analyzed on the actual observed data.

### Data and safety monitoring

An independent data monitoring committee (DMC) will be established comprising several experts from different departments. They will review the safety data and evaluate the quality of the overall study every 6 months.

The detailed procedure of monitoring is described in the DMC charter. There is no interim analysis for this trial. To protect the interests of the subjects, ensure the quality of the trial, and avoid unnecessary financial costs, the trial will be terminated by the sponsor in the following cases:

I. Serious safety issues related to the investigational product, suggesting that the subject is clearly intolerant
II. Significant errors in the clinical trial protocol or major deviations in implementation that make it impossible to evaluate the effects of the drug
III. The State Drug Administration or the ethics committee terminating the trial for certain reasons
IV. Other reasons are not known at this time

### Protocol amendments

As the subjects are all inpatients, investigators ascertain that they will apply due diligence to avoid protocol deviations. Dedicated staff will be assigned to follow-up, remind patients of their drug infusion, evaluate condition status, record outcomes, and provide feedback to the subjects to improve protocol adherence.

Any deviations from the program steps should be documented and explained in the source documents. Any significant deviations that may affect trial sample management, subject safety, or the assessment of safety, efficacy, and tolerability parameters will be promptly reported to the sponsor and the ethics committee.

If the investigator feels a protocol deviation would improve the conduct of the study, this must be considered a protocol amendment, and unless such an amendment is approved by the sponsor and the ethics committee, it cannot be implemented. The sponsor will be responsible for conveying the modification of the protocol to the relevant parties.

### Data confidentiality

To protect the privacy of the trial participants, information about study subjects will be kept confidential. The participants will be identified by their assigned screening numbers and random numbers. Patient’s original medical records are available to authorized representatives of the sponsor and regulatory agencies, and their personal information will not be public unless the applicable laws require.

## Discussions

MSCs inhibit the progression of aGVHD through their immunomodulating activities, and many clinical studies have investigated the potential of MSCs as a treatment option for steroid-refractory aGVHD. However, most of the data are from retrospective or uncontrolled trials with heterogenous indications and procedures [17, 21]. We designed a multicenter, double-blind, randomized, placebo-controlled, phase II study of the umbilical cord-derived mesenchymal stem cell product hUC-MSC PLEB001 for steroid-refractory aGVHD. This study aims to answer two important questions: whether the umbilical cord-derived MSC product hUC-MSC PLEB001 is tolerable and safe in patients with aGVHD after allo-HSCT and whether patients suffering from steroid-refractory aGVHD can benefit from MSC-based treatment.

Most clinical trials focus on steroid-resistant aGVHD; on the other hand, steroid-dependent aGVHD, which is experienced by approximately 30% of patients with aGVHD [22], is ignored. Steroid-dependent aGVHD is associated with morbidity and a prolonged health care burden. In this study, we planned to enroll patients with steroid-refractory aGVHD, which includes both steroid-resistant aGVHD and steroid-dependent aGVHD. Therefore, this strategy of subjective enrollment makes the current trial more comprehensive and practical in the clinical setting.

Bone marrow is the most common source of MSCs in both academic research and commercial application [17, 23]. However, the invasive procedure of bone marrow collection limits its clinical application. Recently, umbilical cord-derived MSCs have gained more attention for their simple availability and low immunogenicity [24]. With potential immunomodulatory activity and stability similar to those of BM-derived MSCs [25], umbilical cord-derived MSCs have been shown to promote engraftment and reduce the incidence of cGVHD [26].

To date, no therapy has yet been established as an optimal second-line treatment for aGVHD. Mesenchymal stem cells have shown substantially promising results in the last two decades. However, solid evidence from high-quality studies is still lacking. This clinical trial will provide additional high-quality evidence for the clinical application of MSCs in the future.

### Trial status

The protocol version number is 6.0, developed on May 31, 2021. This trial opened to recruitment in August 2020. At the time of submission, this trial has recruited 78 participants. Recruitment will be completed on approximately August 31, 2022.

### Study sites

The First Medical Center of Chinese PLA General Hospital; the Fifth Medical Center of Chinese PLA General Hospital; Institute of Hematology & Blood Disease Hospital, Chinese Academy of Medical Sciences & Peking Union Medical College; Shandong Provincial Hospital Affiliated to Shandong First Medical University; the Second Hospital of Hebei Medical University; the First Affiliated Hospital of Zhengzhou University; Henan Provincial People’s Hospital; and the Affiliated Hospital of Xuzhou Medical University.

## Supplementary Information

**Additional file 1. **Interventional Trials (SPIRIT) 2013 checklist.

## Data Availability

All data and results of this clinical trial belong to the sponsor and should not be provided to a third party without the permission of the sponsor, except as required by the regulatory authority.

## Abbreviations

AE: Adverse event
aGVHD: Acute graft-versus-host disease
allo-HSCT: Allogeneic hematopoietic stem cell transplantation
ChiCTR: Chinese Clinical Trial Registry
CHM test: Cochran–Mantel–Haenszel Test
CIMS-CRS: Centralized Randomization System of Clinical Information Management System
CMV: Cytomegalovirus
CR: Complete response
CRF: Case report form
CRP: C-reactive protein
CTCAE: Common Terminology Criteria for Adverse Event
DCR: Durable Complete Response
DMC: Data monitoring committee
EC: Ethics committee
ECOG: Eastern Cooperative Oncology Group
eCRF: Electronic case report form
EDC: Electronic data management center
FDA: Food and Drug Administration
GCP: Good Clinical Practice
HBV: Hepatitis B virus
HCV: Hepatitis C virus
HIV: Human immunodeficiency virus
HLA: Human leucocyte antigen
ICF: Informed consent form
ICTRP: International Clinical Trial Registration Platform
IDO: Indoleamine 2,3-dioxygenase
JAK: Janus kinase
MSC: Mesenchymal stem cell
NCI: National Cancer Institute
NO: Nitric oxide
NR: No response
NYHA: New York Heart Association
ORR: Overall response rate
OS: Overall survival
PR: Partial response
PD: Progression of disease
RCT: Randomized controlled trial
SAE: Serious adverse events
SAS: Statistical Analysis System
TP: Treponema pallidum
Treg cell: Regulatory T cells
Th17 cell: T helper cell 17
WOCF: Worst observation carried forward
